# Impact of COVID-19 pandemic and related isolation measures on violence against children in Egypt

**DOI:** 10.1186/s42506-021-00071-4

**Published:** 2021-04-20

**Authors:** Seham Ahmed AboKresha, Elsayed Abdelkreem, Rasha Abd Elhameed Ali

**Affiliations:** 1 Public Health and Community Medicine Department, Sohag University, Sohag, Egypt; 2 Department of Pediatrics, Sohag University, Sohag, Egypt

**Keywords:** Violence against children, Child maltreatment, COVID-19, Isolation measures, Post-traumatic stress disorder

## Abstract

**Background:**

Coronavirus disease 2019 (COVID-19) and related isolation measures have substantial adverse economic, social, and psychological consequences and expose children to increased risk of violence. The present study aimed to investigate the impact of the COVID-19 pandemic on violence against children in Egypt.

**Methods:**

An online survey, in Arabic, was disseminated during the period from 9 to 13 April 2020, to parents of children who were up to 18 years old residing in Egypt, selected using a snowball sampling technique, during the period from 25 March to 8 April during the implementation of the nationwide compulsory isolation measures against COVID-19 (25 March to 8 April 2020). The survey covered three areas: socio-demographic data, psychological impact measured using the Impact of Event Scale-Revised (IES-R), and violence against children during the past 2 weeks measured using a modified parent-report of a child abuse screening tool (ICAST-P) developed by the International Society for the Prevention of Child Abuse and Neglect.

**Results:**

Out of 1118 completed survey responses, 90.5% of children were subjected to violent discipline, 88.7% experienced psychological aggression, and 43.2% encountered severe physical punishment. Approximately 60% of respondents reported a moderate-to-severe psychological impact (IES-R scores ≥ 33), which was associated with a higher rate of violent discipline (OR: 9.3; 95% CI: 5.37–16.027; *p* < 0.001).

**Conclusions:**

This is the first study in Egypt to provide evidence on the association of COVID-19 pandemic, its psychological impact, and increased rates of violence against children. Effective multilevel strategies are urgently required to protect children from violence and its catastrophic consequences during the continually evolving COVID-19 pandemic.

## Introduction

Coronavirus disease 2019 (COVID-19) is an emerging and rapidly evolving major public health challenge for the entire global population. COVID-19 was first reported in December 2019 in Wuhan City (Hubei Province, China) and has been rapidly spreading to affect millions worldwide. Beyond the health crisis, COVID-19 has major adverse economic, social, and psychological consequences [1, 2].

To limit the spread of COVID-19, countries have implemented variable control measures up to complete lockdown, including school closures [3]. The United Nations Educational, Scientific and Cultural Organization (UNESCO) estimates that over 1.5 billion children are out of schools and childcare centers because of COVID-19 lockdown [4]. Consequently, families have been confined at home with their children. These circumstances have had negative psychological effects and exposed the children to an increased risk of violence with its potential adverse consequences on their future life [5–8].

Violence against children, encompassing all forms of violence against individuals under 18 years of age, is an important public health, human right, and social concern. Child maltreatment includes physical, psychological, and sexual abuse and neglect by parents or other legal caregivers. A systematic review in 2016 estimated that up to 1 billion children aged 2‑17 years’ experience different forms of violence every year globally [9]. Children exposed to violence may suffer from significant lifelong adverse consequences, which extend to families, societies, and countries [10, 11]. Under the United Nations Convention on the Rights of the Child, children all over the world have the right to be safe from violence. The 2014 Egyptian Constitution and Article 1 of the 2008 Egyptian Child Law guaranteed that right for children [12]. In Egypt, a 2014 UNICEF study on violence committed against children in Cairo, Alexandria, and Assiut found that two-thirds of youngsters within the areas covered had been subjected to physical abuse, and 78% were victims of emotional violence [12].

Previous studies reported increased rates of psychological distress and violence against children during wide-scale health emergencies, including outbreaks of infections, associated with school closure and social isolation [13]. To date, there has been no adequate information on violence against children during the COVID-19 pandemic [6, 13].

Timely knowledge and understanding of the impact of the COVID-19 pandemic and related isolation measures on violence against children are urgently required to provide updated information for the society, healthcare system, and national authorities [6, 8]. The aim of the present study was to identify the magnitude, pattern, and risk factors of violence against children and its association with parents’ psychological stress during the period of the isolation measures related to the COVID-19 pandemic in Egypt.

## Methods

### Study Setting, design, and participants

**Fig. 1:**
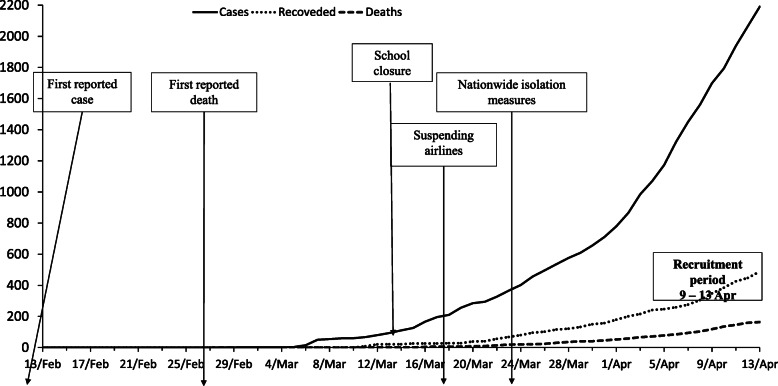
National trend of the 2019 coronavirus disease (COVID-19) outbreak in Egypt from 13 February to 13 April 2020. The recruitment of participants took place from 9‑13 April

Egypt is a developing country with approximately 100 million inhabitants living in an area of 1.01 million km^2^. The national trend of the COVID-19 epidemic in Egypt is illustrated in Fig. 1. Since the identification of the first case of COVID-19 in Egypt on February 13, 2020, the numbers of confirmed cases, convalescent individuals, and deaths attributed to COVID-19 infection have continued to increase, with a significant rise since the beginning of April 2020. COVID-19 has affected all age groups from all Egyptian areas, but most deaths were among elderly people with medical comorbidities. National precautionary actions against COVID-19 in Egypt started by school closure (March 15, 2020) and extended to nationwide lock down (March 25, 2020) [14].

This cross-sectional study was conducted between February and April 2020. Eligible participants were Egyptian parents of children up to 18 years old who were residing in Egypt during the first 2 weeks of implementing the nationwide compulsory isolation measures against COVID-19 (from 25 March to 8 April 2020). We adopted a snowball sampling technique to recruit potential participants.

The sample size was calculated using the Epi Info program developed by Centers for Disease Control and Prevention (CDC) in Atlanta, Georgia (US) version7 software, based on the following assumptions: a 68% prevalence of violence against children, as reported by a 2014 UNICEF study [12]; 95% level of confidence and 5% precision; and 1 as design effect 1. The calculated minimum sample size was 364. For proper representation of the Egyptian community, recruitment was opened for 2 weeks to maximize the sample size. A total of 1191 responses were submitted to the web-based survey, but missing data led to the exclusion of 73 responses. Ultimately, 1118 completed questionnaires were included in the analysis. We received 42 completed responses on the first day, 384 on the second day, 435 on the third day, 251 on the fourth day, and only 6 on the fifth day. The average time to complete the survey was 18 min.

### Data collection method

We used a web-based methodology for survey design, dissemination, and data collection because the Egyptian government imposed compulsory nationwide isolation measures and asked the public to minimize direct personal contact and isolate themselves at home. We prepared an anonymous electronic survey using the Microsoft forms (http://forms.office.com) and distributed it to the general Egyptian population using multiple means of communication and social media, such as Facebook, Twitter, Instagram, and WhatsApp. The web-based survey was also sent to university students who were encouraged to disseminate it to other people (friends, family members, and relatives). Potential participants for this study were invited by other study respondents through an electronic link to the survey. Respondents completed the anonymous electronic questionnaire in Arabic. Data collection occurred over 5 days (9–13 April 2020).

### Validation of the study tools

The psychological impact of COVID-19 on survey respondents was assessed using the Impact of Event Scale-Revised (IES-R), and violence against children was assessed by the parent-report version of the International Society for the Prevention of Child Abuse and Neglect (ISPCAN) child abuse screening tool (ICAST-P) [15]. Both questionnaires were translated into Arabic according to the standard guidelines of translation and cultural adaptation of psychological and educational tests [16]. First, forward translation was carried out by two independent bilingual, bicultural professional translators who are native Arabic speakers. A consensus version of the scale was reached, which was reviewed by external experts. Second, back translation of the first translated version into English was performed by another bilingual, bicultural professional translator who is a native English speaker (with no access to the original English version), followed by comparison with the original English source to produce the second translated version. Third, face validity was conducted through an online cognitive interview with 50 potential Egyptian participants of different educational levels to evaluate understanding, interpretation, and appropriateness of each item. Reliability of both questionnaires was tested using the Cronbach’s alpha. The Cronbach’s alpha coefficient was 0.89 for IES-R and 0.88 for the adopted ICAST-P.

### Survey development

The survey covered three areas: socio-demographic data, the psychological impact of COVID-19 pandemic on parents, and violence against children during the study period.

Socio-demographic data included respondents’ gender, age, marital status, educational level, residence, employment status, monthly income, and number of children and whether the children had chronic diseases.

The psychological impact of COVID-19 on survey respondents was assessed using IES-R, which measures the subjective response of adults to a certain potentially stressful life event. IES-R is an internationally validated, non-cultural specific, self-administered questionnaire, which is composed of 22 items on a five-point Likert scale (where 0 = not at all, and 4 = extremely) with three subscales: intrusion (inability to keep memories of the event from returning), avoidance (an attempt to avoid stimuli and triggers that may bring back those memories), and hyper arousal (a primary symptom of post-traumatic stress disorder (PTSD) that occurs when a person’s body suddenly kicks into high alert as a result of thinking about their trauma). This instrument (IES-R) is designed to capture the American Psychiatric Association Diagnostic and Statistical Manual of Mental Disorders criteria for PTSD [17]. We used a cut-off score of ≥33 because it was reported to have good diagnostic power (0.88) for PTSD [18].

Violence against children was assessed by the Arabic parent-report version of ICAST-P [15]. The original ICAST-P asks parents about disciplinary practice and omission of care (non-violent, moderate physical, severe physical, psychological, neglect, and sexual abuse) toward a randomly selected index child under 18 years. It measures lifetime and past-year abusive exposure and neglect (once a week or more, several times a month, about once a month, several times a year, once or twice a year, not in the past-year, or never); hence, it was not appropriate in the present time-sensitive study. To overcome the long reporting time-frame, Meinck et al. previously designed ICAST-Trial to estimate the actual number of abusive incidents in the past month (0, 1, 2, 3, 4, 5, 6, 7, or 8+) for evaluating the effectiveness of parenting programs [19]. We adopted a similar approach through changing the response codes and reporting time-frame to estimate the actual number of violent incidents (0 was assigned to not at all; 1, to once; 2, to 2–3 times; 3, to 4–7 times; and 4, to >7 times) in the past 2 weeks. The neglect subscale and two items of the psychological discipline subscale (locking out of the house and public humiliation) were excluded because of contextual consideration (COVID-19 pandemic and related isolation measures). During the step of face validity (interviewing nine potential study participants online), two items of sexual abuse were considered inappropriate and unacceptable; therefore, they were also excluded. The final modified version of ICAST-P used in this study included 35 items with four subscales: non-violent discipline (5 items), moderate physical discipline (12 items), severe physical discipline (8 items), and psychological discipline (10 items). In addition, there were general questions on the index child’s age, gender, birth order, and educational level.

### Ethical approval

The study was approved by the Research Ethics Committee of the Faculty of Medicine, Sohag University, and was carried out in accordance with the principles of the 1964 Declaration of Helsinki and its 2013 revision. Informed consent was obtained from the respondents by submitting the consent statement included in the survey.

### Statistical analysis

Survey data were analyzed using the IBM SPSS® software version 20 (IBM SPSS Statistics, New York, USA). Descriptive statistics were presented as frequencies and percentages for categorical variables and mean ± standard deviation for continuous variables. Chi-squared and odds ratio tests and multivariate logistic analysis were used for calculating the association between socio-demographic parameters, the psychological impact (using an IES-R cut-off score ≥ 33), and violent discipline. All calculations were based on alpha 5% and beta 80%. A *p* value (two-tailed) < 0.05 was considered to be statistically significant.

## Results

Descriptive statistics of the study variables are presented in Tables 1 and 2**.** Participants’ ages ranged between 20 and ≥ 50 years; most respondents were mothers (88.1%), who were currently married (95.3%), urban residents (80.6%), employed at a government agency (66.3%), well-educated (bachelors and postgraduate, 97.7%), and had monthly income ≤3000 Egyptian pounds (53.1%). Most of them (72.3%) had two to three children, with no chronic medical conditions among children (93.5%) and no other persons living with the family in the same household (65.6%). Most of the included index children were males (60.6%), aged 2–12 years (75.7%), of first birth order (79.7%), and in kindergarten/primary school (69.8%). As listed in Table 1, rates of reported violent discipline among parents were significantly associated with the female gender, age 30 to 50 years, urban residence, a family income ≥ 3000 Egyptian pounds, less children, and presence of other individuals living with the family. Certain socio-demographic characteristics were associated with an increased risk of PTSD among parents, including the female gender and age groups 20 to <50 years with private work and a monthly income ≤ 3000 Egyptian pounds, as presented in Table 1.

**Table 1:** Socio-demographic characteristics of survey respondents and their association with psychological impact of the pandemic on the parents and violent discipline against their children during the COVID-19 pandemic in Sohag governorate, Egypt, 2020

Parameters	Parents respondents ***N*** = 1118 (%)	Parents’ IES-R score ≥ 33 ***N*** = 646 (%)	OR	***p***	Parents’ violent discipline^**#**^ ***N*** = 1012. (%)	OR	***p***
***Parents’ gender***							
Male ®	133 (11.9)	60 (45.1)			108 (81.2)		
Female	985 (88.1)	586 (59.5)	0.56	0.002	904 (91.8)	0.387	0.00
***Parents’ age (years)***		**OR = 0.791** ***p*** **= 0.009**			**OR = 0.917** ***p*** **= 0.557**		
20 to <30	228 (20.4)	144 (63.5)	2.952	0.001	193 (84.6)	1.991	0.064
30 to <40	752 (67.2)	429 (57.0)	2.287	0.007	703 (93.5)	5.181	0.000
40 to <50	89 (8.0)	55 (61.8)	2.786	0.005	80 (89.9)	3.210	0.015
≥50 ®	49 (4.4)	18 (36.7)			36 (73.5)		
***Parents’ marital status***		**OR = 1.23** ***p*** **= 0.322**			**OR = 73 × 10**^**6**^ ***p*** **= 0.997**		
Married	1,065 (95.3)	614 (57.7)	0.38	0.09	959 (90.0)	0.00	0.99
Divorced	35 (3.1)	18 (51.4)	0.30	0.07	35 (100)	1.00	1.00
Widow/er ®	18 (1.6)	14 (77.8)			18 (100)		
***Parents’ residence***		**OR = 0.804** ***p*** **= 0.151**			**OR = 0.467** ***p*** **= 0.001**		
Urban	901 (80.6)	530 (58.8)	1.244	0.151	829 (92.0)	2.139	0.001
Rural	217 (19.4)	116 (53.5)			183 (84.3)		
***Parents’ employment***		**OR = 1.17** ***p*** **= 0.087**			**OR = 0.995** ***p*** **= 0.973**		
No ®	285 (25.5)	157 (55.1)			256 (89.8)		
Government agency	741 (66.3)	425 (57.4)	1.09	0.51	674 (91.0)	1.14	0.57
Private sector	49 (4.4)	39 (79.6)	3.18	0.00	44 (89.8)	0.99	0.99
Freelancer	43 (3.8)	25 (58.1)	1.13	0.71	38 (88.4)	0.86	0.77
***Parents’ highest education***		**OR = 0.828** ***p*** **= 0.080**			**OR = 0.95** ***p*** **= 0.779**		
Elementary	8 (0.7)	8 (100.0)	128 × 10^6^	0.999	8 (100%)	180 × 10^6^	0.999
High school	18 (1.6)	9 (50.0)	0.790	0.622	13 (72.2)	0.291	0.023
Bachelors	505 (45.2)	301 (59.6)	1.165	0.214	463 (91.7)	1.232	0.325
Postgraduate ®	587 (52.5)	328 (55.9)			528 (89.9)		
***Parents’ income (LE)***		**OR = 0.69** ***p*** **= 0.003**			**OR = 2.54** ***p*** **< 0.001**		
≤3000	594 (53.1)	368 (62)	1.441	0.003	517 (87)	0.393	0.00
˃3000	524 (46.9)	278 (53.1)			495 (94.5)		
***Number of children***		**OR = 1.11** ***p*** **= 0.327**			**OR=2.51** ***p*** **< 0.001**		
1®	207 (18.5)	116 (56.0)			163 (78.7)		
2–3	808 (72.3)	466 (57.7)	1.069	0.671	756 (93.6)	3.924	0.000
4–5	89 (8)	55 (61.8)	1.269	0.358	79 (88.8)	2.133	0.044
>5	14 (1.3)	9 (64.3)	1.412	0.548	14 (100.0)	436 × 10^6^	0.999
***Children with chronic disease***		**OR = 0.743** ***p*** **= 0.239**			**OR = 0.687** ***p*** **= 0.430**		
Yes	73 (6.5)	47 (64.4)	1.35	0.239	68 (93.2)	1.455	0.43
No®	1045 (93.5)	599 (57.3)			944 (90.3)		
***Other individuals living with family***		**OR = 0.815** ***p*** **= 0.110**			**OR = 1.88** ***p*** **= 0.002**		
Yes	385 (34.4)	235 (61.0)	1.22	0.11	334 (86.8)	0.531	0.002
No ®	733 (65.6)	411 (56.1)			678 (92.5)		
**Total**	**1118**	**646 (57.8)**			**1012 (90.5)**		

®Reference category

**Table 2:** Socio-demographic characteristics of children exposed to different types of violent discipline during the COVID-19 pandemic in Sohag governorate, Egypt, 2020

Parameters	Index children ***N*** = 1118. (%)	Violent discipline ***N*** = 1012 (%)	OR	***p***	Severe physical discipline ***N*** = 483 (%)	OR	***p***	Psychological discipline ***N*** = 992 (%)	OR	***p***
***Child gender***		**OR = 1.35** ***p*** **= 0.162**			**OR = 0.527** ***p*** **< 0.001**			**OR = 1.79** ***p*** **= 0.005**		
Male	678 (60.6)	607 (59.9)	0.739	0.16	334 (69.1)	1.90	0.00	587 (59.1)	0.55	0.005
Female ®	440 (39.4)	405 (40.1)			149 (31.9)			405 (40.9)		
***Child age*** **(*years*)**		**OR = 1.28** ***p*** **< 0.001**			**OR = 0.80** ***p*** **< 0.001**			**OR = 1.23** ***p*** **= 0.007**		
<2	149 (13.3)	110 (10.8)	0.452	0.013	65 (13.4)	1.799	0.022	105 (10.5)	0.38	0.002
2–<5	286 (25.6)	271 (26.7)	2.89	0.004	148 (30.6)	2.493	0.000	271 (27.3)	2.89	0.004
5–<8	236 (21.1)	223 (22)	2.75	0.009	127 (26.2)	2.708	0.000	218 (21.9)	1.94	0.064
8–<12	324 (29)	302 (29.8)	2.20	0.021	106 (21.9)	1.130	0.594	292 (29.4)	1.46	0.235
≥ 12	123 (11)	106 (10.4)			37 (7.6)			106 (86.2)		
***Child birth order***		**OR = 0.911** ***p*** **= 0.561**			**OR = 0.66** ***p*** **= 0.000**			**OR = 1.05** ***p*** **= 0.758**		
1 ®	891 (79.7)	812 (80.2)			403 (83.4)			792 (79.8)		
2–3	158 (14.1)	136 (13.4)	0.601	0.04	63 (13)	0.810	0.232	136 (13.7)	0.77	0.309
4–5	50 (4.5)	45 (4.4)	0.876	0.78	14 (2.8)	0.475	0.021	45 (4.5)	1.12	0.807
>5	14 (1.3)	14 (1.3)	157 × 10^6^	0.99	3 (0.6)	0.000	0.998	14 (1.4)	201 × 10^6^	0.999
***Child education level***		**OR = 1.66** ***p*** **< 0.001**			**OR = 0.52** ***p*** **= 0.001**			**OR = 1.60** ***p*** **< 0.001**		
No ®	218 (19.5)	170 (16.7)			86 (17.8)			165 (16.6)		
Kindergarten	345 (30.9)	321 (31.7)	3.8	0.00	198 (11.8)	2.06	0.00	316 (31.8)	3.50	0.000
Primary school	435 (38.9)	418 (41.3)	6.9	0.00	161 (33.3)	0.90	0.55	408 (41.1)	4.85	0.000
Prep. school	99 (8.9)	82 (8.1)	1.3	0.00	34 (7)	0.80	0.39	82 (8.2)	1.54	0.157
High school	21 (1.9)	21 (2.1)	456× 10^6^	0.99	4 (0.8)	0.36	0.08	21 (2.1)	518 × 10^6^	0.998
**Total**	**1118**	**1,012 (90.5%)**			**483 (43.2%)**			**992 (88.7%)**		

®Reference category

Regarding the psychological impact of COVID-19, the mean IES-R score of survey respondents was 35.65 (±12.92). More than half of the respondents (57.8%) had scores ≥ 33, indicating a moderate-to-severe psychological impact, which is consistent with PTSD (Table 2). Among the studied children, male children, those aged <2, 2–5, and 5–8 years; those with a birth order of 2–3; and those with kindergarten, primary, and preparatory levels of education, were more exposed to violence.

**Fig. 2:**
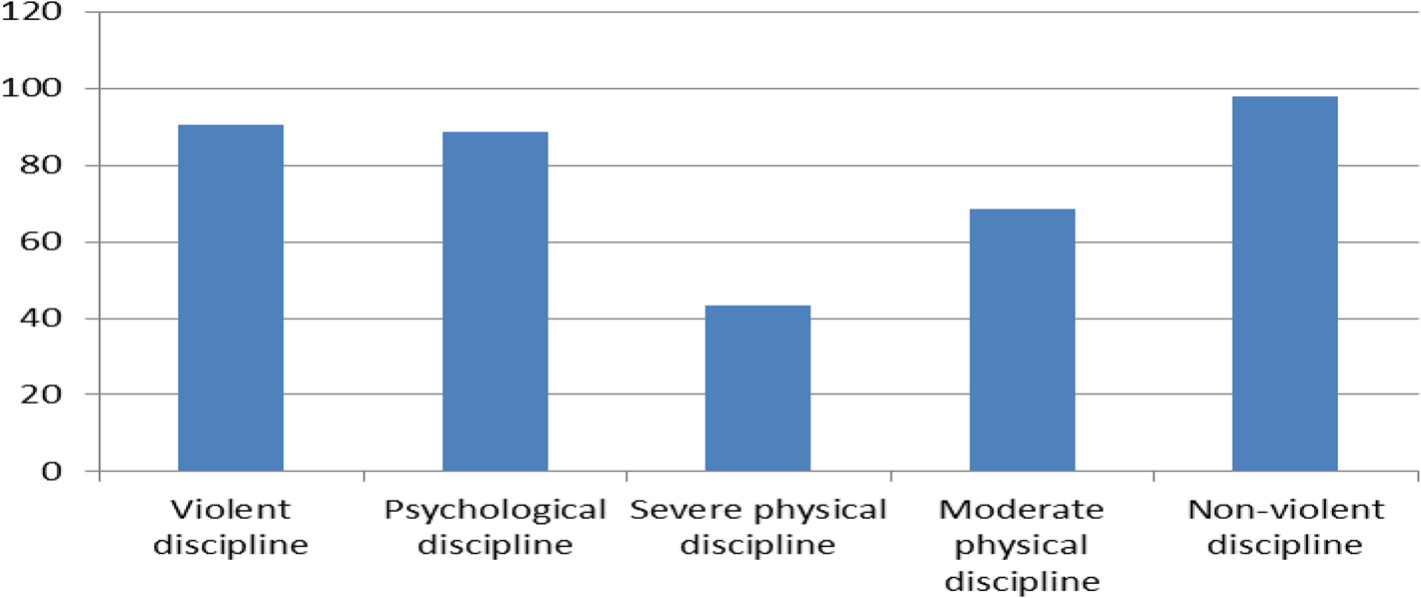
Distribution of studied children according to the discipline practice reported by their parents during COVID-19 pandemic, Egypt, 2020, using adapted ICAST-P

As shown in Table 3 and Fig. 2, the majority of children (90.5%) were subjected to at least one form of violent discipline in the past 2 weeks, 88.7% experienced psychological aggression, and 43.2% underwent severe physical punishment. Non-violent discipline was practiced by most participants. For detailed information of each subcategories of violent discipline, see Table 3.

**Table 3:** Reported discipline practice of parents toward their children during COVID-19 pandemic using adapted ICAST-P in Sohag governorate, Egypt, 2020

Practice	Respondents (%) (Total no. = 1118)
***Non-violent discipline***	**1094 (97.9)**
1. Explained	308 (28.2)
2. Told to stop something	312 (28.5)
3. Time out	247 (22.5)
8. Distracted	127 (11.6)
23. Took away privileges	100 (9.2)
***Moderate physical discipline***	**768 (68.7)**
4. Shook child (>2 years)	193 (25.2)
6. Hit on buttocks w/object	99 (12.9)
7. Hit elsewhere w/object	85 (11)
9. Twisted ear	84 (10.9)
10. Knuckled back of head	77 (10)
11. Pulled hair	83 (10.8)
16. Others^a^	0
17. Painful kneel/stand	20 (2.6)
19. Spanked on buttocks w/bare hands	33 (4.3)
20. Spanked elsewhere w/bare hands	45 (5.9)
25. Pinched	26 (3.5)
26. Slapped face/back of head	23 (2.9)
***Severe physical discipline***	**483 (43.2)**
5. Shook child (< 2 years)	135 (28)
15. Kicked	89 (18.4)
21. Choked	76 (15.7)
30. Smother	58 (12)
31. Others^a^	0
32. Beat up	64 (13.3)
33. Threaten with knife or gun	61 (12.6)
35. Others^a^	0
***Psychological discipline***	**992**^**#**^ **(88.7)**
12. Threatened to abandon	134 (13.5)
13. Shouted	181 (18.2)
14. Threatened to invoke spirits	107 (10.8)
18. Cursed	23 (2.3)
22. Threatened to send away	121 (12.2)
24. Insulted	151 (15.3)
27. Refused to speak	51 (5.2)
28. Withhold food	103 (10.3)
29. Told you wished he/she never born	100 (10.1)
34. Lock in a dark room	21 (2.1)
**Violent discipline**	**1012 (90.5)**

^a^Chili pepper mouth, burn, and gave drug or alcohol

^**#**^The sum does not add to 100 because of multiple responses

Table 4 displayed the final model of logistic regression analysis of the psychological impact of COVID-19 on parents and practicing different types of violent discipline against their children after adjustment of other socio-demographic variables. It shows that there was a strong association between psychological impact of COVID-19 pandemic on parents and practicing violence against their children. Children to parents with probable PTSD were nine times more exposed to violent discipline than those to parents with no/mild psychological impact.

**Table 4:** Final model logistic regression analysis of psychological impact related to COVID-19 pandemic and violent discipline of parents against their children in Sohag governorate, Egypt, 2020

	IES- R score		***p*** value	OR (95% CI)
	< 33	≥ 33		
**Participants**	472 (42.2)	646 (57.8)		
**Violent discipline**	382 (80.9)	630 (97.5)	***p*** **<0.001**	OR = 9.3 (95% CI 5.4‑16.0)
**Severe physical discipline**	136 (28.8)	347 (53.7)	***p*** **<0.001**	OR = 2.9 (95% CI 2.3‑3.7)
**Psychological discipline**	377 (79.9%)	615 (95.2%)	***p*** **<0.001**	OR = 5.0 (95% CI 3.3‑7.7)

## Discussions

This is the first study in Egypt to investigate the impact of the COVID-19 pandemic and related isolation measures on violence against children. Through surveying 1118 parents who were residing in Egypt between March 25 and April 8, 2020, we found a high rate (90.5%) of violent discipline with more than two-thirds of children experiencing severe physical violence. More than half of the respondents reported a moderate-to-severe psychological impact of COVID-19, which was significantly associated with a higher rate of violent discipline. These alarming findings warrant a timely comprehensive strategy to support families and protect children from violence during the continually evolving COVID-19 pandemic.

Evidence demonstrates that children are at increased risk of violence during times of public health emergencies [8]. Several reports during the current COVID-19 pandemic suggested that child abuse was exacerbated during this time of economic uncertainty and parents stress. In Jianli County (Hubei province, China), incidences of domestic violence reported to the police tripled during the lockdown in February: from 47 in 2019 to 162 in 2020 [20]. Fabbri et al., in their study that estimated the anticipated effect of COVID-19 on violent discipline against 1–14-year-old children at home using multivariable predictive regression models, reported that in Nigeria, Mongolia, and Suriname, under a “high restrictions” scenario, there would be a 35–46% increase in violent discipline scores, and under a “lower restrictions” scenario, there would be a 4–6% increase in violent discipline scores [21].

Results of the current study indicated that 90.5% of the studied children were subjected to violent disciplines. This rate is much higher than the results of a UNICEF study conducted in 2014, wherein 68.5% of the studied children were exposed to violent disciplines, [12] despite the reporting time-frame being markedly shorter (2 weeks) in the present study compared to the UNICEF study (3 months). Our results are also higher than the results of a meta-analysis of 38 reports from 96 countries on past-year prevalence of violence against children that indicated that a minimum of 50% or more of children in Asia, Africa, and Northern America experienced past-year violence [9].

However, a recent systematic review undertaken in 2019, of data of physical and emotional child maltreatment, sexual abuse, bullying and fighting, and violence in schools, retrieved using national population-based surveys, demonstrated that physical, sexual, and emotional violence against adolescents is widespread in the Arab region and that in many studies, the prevalence rates exceeded other regional or global estimates, including high rates of violent discipline [22]. The higher rate of violent disciplines in the current study may be explained by the fact that the aforementioned studies were conducted during usual conditions and were not related to a pandemic that necessitated lockdown. These high baseline rates of violence are alarming and are expected to increase during the COVID-19 pandemic. Studies conducted after the pandemic indicated that the rate of violent disciplines was increased during the pandemic in many countries [23], for example, in China, Xue et al. [23] analyzed over 1 million tweets related to family violence and COVID-19 from April 12 to July 16, 2020. Examining the data form the child helplines during the first 6 months of 2020, Petrowski et al. demonstrated that overall, the number of contacts to helplines in the USA seemed to have drastically increased since the beginning of the pandemic. However, they concluded that the number of contacts related to violence had increased in some countries, whereas it had decreased in others [24].

The present study identified certain socio-demographic risk factors of violence against children among parents and children. Poor parental education, lower family income, and small number of children are frequently reported as risk factors of violence against children. Our results are in agreement with these studies [9, 25]. Our results are also in accordance with the research indicating that boys and girls are at equal risks of physical and psychological abuse, with a higher risk of sexual abuse for girls [9]; that boys are more exposed to violent discipline, particularly severe physical punishment; and that children under 4 years and adolescents are subject to a higher rate of violence that increases with age [9, 15, 22]. It is worth highlighting that violence against children is a multifactorial problem at levels, of not only individuals (parents and children) but also close relationships, community, and society [9, 22].

In the present study, 57.8% of respondents had a moderate-to-severe psychological impact (consistent with PTSD) related to the COVID-19 pandemic. These findings are in agreement with those in other studies; two studies in China reported PTSD in 53.8% and 7.6% of participants during the COVID-19 pandemic [26, 27]. PTSD was previously reported in 25.8% of respondents during the 2003 severe acute respiratory distress (SARS) outbreak in Singapore [28]. Moreover, a study that assessed the effect of the COVID-19-related lockdown on Tunisian women’s mental health and gender-based violence indicated that more than half of the participants (57.3%) reported extremely severe distress symptoms [29]. The higher rates of PTSD in our study, despite the significantly lower official number of cases and deaths related to COVID-19 in Egypt, may be attributed to differences in the socio-demographic background, pronounced impact of isolation measures, and higher public fears of infection and its adverse social and economic consequences, which might be exaggerated by the mass media and the deficiency of ventilators and intensive care facilities.

The identified risk factors of PTSD in this study are generally consistent with those in previous studies. Females and young people were reported to have a higher risk of PTSD [7, 26, 30]. In a Canadian study, participants with a lower income had a significantly higher rate of PTSD related to the SARS outbreak [30]. Individuals with lower income are more likely to be affected by any temporary decrease in income. Workers in the private sector appear to be more exposed to financial loss and redundancy compared to the more secure jobs in government agencies [7].

The present study demonstrated a strong association between the adverse psychological impact of the COVID-19 pandemic in parents and rates of violence against children. Children of parents with COVID-19-related PTSD were nine times more likely to be exposed to violence than those of parents with no or mild psychological impact. Based on previous reports of increased rates of PTSD with prolonged periods of quarantine [7], the magnitude of violence against children may markedly escalate with the protracted duration of the isolation measures associated with the COVID-19 pandemic.

Despite the challenges associated with the COVID-19 pandemic and the related isolation measures, some researchers have indicated that this situation can be an opportunity to enhance the relationship between parents and children. During lockdown times, children will benefit from keeping a sound, healthy, and productive daily schedule. Having a sense of aim and routine can help children and adults avoid negative feelings [5, 6, 8]. The findings of this study will contribute to the ongoing efforts of protecting children from violence during the rapidly expanding COVID-19 pandemic in Egypt and the world.

### Limitations

The present study has some limitations. First, we used a snowball sampling strategy because of the limited resources and time-sensitivity of the COVID-19 pandemic. This sampling strategy does not rely on random selection; consequently, the study population does not mirror the actual characteristics of the general population. Second, the use of a web-based method for data collection might have restricted study participation to individuals with certain education and technology skills. This limits the generalizability of study findings, particularly among the less educated population. Third, for ethical reasons and age limits, violence against children was evaluated based on parents’ reports only; children’s reports were not studied. Fourth, it would have been ideal to carry out a follow-up study on the same study population to provide stronger evidence, investigate the effectiveness of intervention programs, and study the potential psychosocial impact of violence on the children. However, this is not applicable given the ethical mandate of anonymity and confidentiality. Fifth, the data were collected after only 1 month of the lockdown, and the expectation is that the rate of violence will escalate with prolonged lockdown and school closure. Finally, the pattern of violence described cannot be attributed to COVID-19 in view of the absence of information regarding the child disciplinary pattern prior to the pandemic.

## Conclusions

This study provides an insight into the magnitude, characterization, and risk factors of violence against children during the COVID-19 pandemic in Egypt. The vast majority of the studied parents (90.5%) reported practicing violent discipline against their children during the COVID-19 lockdown, shedding light on an alarming situation. Poor parental education, lower family income, and a large number of children were significant risk factors of violence against children. Notably, more than half of the studied parents experienced a moderate-to-severe psychological impact (consistent with PTSD) related to the COVID-19 pandemic, which was strongly associated with violence against children. Children of parents with COVID-19-related PTSD were nine times more likely to be exposed to violence than those of parents with no or mild psychological impact. These findings indicate the urgent need for increasing the public awareness regarding healthy parenting discipline and the best use of childhood time at home, as well as setting up a secure and easy system for parental counseling. Providing the parents with psychological support during this pandemic and training of healthcare professionals to regular screening for child abuse and neglect are highly recommended particularly those with poor socioeconomic status, to overcome the burden of the COVID-19 pandemic and isolation measures.

## Data Availability

All data are available on demand from the corresponding author.

## Abbreviations

CDC: Centers for Disease Control and Prevention
COVID-19: Coronavirus disease 2019
ICAST-P: Child Abuse Screening Tool-Parents
IES-R: Impact of Event Scale-Revised
ISPCAN: International Society for the Prevention of Child Abuse and Neglect
PTSD: Post-traumatic stress disorder
SARS: Severe acute respiratory distress syndrome
UNESCO: United Nations Educational, Scientific and Cultural Organization
UNICEF: The United Nations Children’s Fund
